# Effect of Print Orientation and Thermal Aging on the Flexural Strength of Zirconia-Reinforced Three-Dimensional-Printed Restorative Resin Materials

**DOI:** 10.3390/molecules30112337

**Published:** 2025-05-27

**Authors:** Yunus Emre Özden, Bengü Doğu Kaya, Pınar Yılmaz Atalı, Fusun Ozer, Zeynep Ozkurt Kayahan

**Affiliations:** 1 Department of Prosthodontics, Dentistry Faculty, Yeditepe University, Istanbul 34755, Türkiye; emre.ozden@yeditepe.edu.tr (Y.E.Ö.); zeynep.ozkurt@yeditepe.edu.tr (Z.O.K.); 2 Department of Restorative Dentistry, Dentistry Faculty, Çanakkale Onsekiz Mart University, Çanakkale 17100, Türkiye; bengu.dogukaya@comu.edu.tr; 3 Department of Restorative Dentistry, Dentistry Faculty, Marmara University, Istanbul 34722, Türkiye; pinar.atali@marmara.edu.tr; 4 Department of Restorative Dentistry, Dentistry Faculty, Pennsylvania University, Philadelphia, PA 19104, USA

**Keywords:** 3D printing, flexural strength, print orientation, thermal aging, restorative materials

## Abstract

This study evaluated the effects of print orientation and thermal aging on the flexural strength (FS) and flexural modulus (FM) of novel permanent three-dimensional (3D)-printed polymethyl methacrylate (PMMA) resins reinforced with nano-zirconia and nano-silica. Bar-shaped specimens (25 × 2 × 2 mm) were fabricated using a digital light processing (DLP) 3D printer (Asiga Max UV, Asiga Inc., Australia) in two orientations (0° and 90°). Specimens underwent three-point bending tests at 24 h and after artificial thermal aging (10,000 and 30,000 cycles) to simulate one and three years of intraoral conditions. Scanning electron microscopy (SEM) was used to analyze fracture patterns. Print orientation did not significantly affect FS or FM (*p* > 0.05). However, artificial aging significantly reduced FS and FM after 10,000 cycles (*p* < 0.001), with further deterioration after 30,000 cycles. The micro hybrid resin composite exhibited higher FS than the 3D-printed materials throughout aging. SEM analysis revealed distinct fracture patterns, with 3D-printed resins displaying radial fractures and the micro hybrid composite exhibiting horizontal fractures. These findings indicate that aging plays a more critical role in the long-term mechanical performance of 3D-printed restorative resins than print orientation. This study provides original data on the effects of print orientation and prolonged thermal aging on the mechanical behavior of permanent three-dimensional (3D)-printed dental resins. Furthermore, the comparative evaluation of aging protocols simulating one and three years of intraoral service represents a novel contribution to the existing literature. Further studies are required to optimize the mechanical durability of 3D-printed dental restorations.

## 1. Introduction

Computer-aided design and computer-aided manufacturing (CAD-CAM) techniques, which encompass subtractive manufacturing (SM) and additive manufacturing (AM), have revolutionized the fabrication of dental prostheses. Among these, AM, also known as 3D printing, stands out due to its advantages, such as rapid prototyping, unrestricted shaping capabilities, and efficient material utilization [1]. Initially, additive manufacturing was primarily used for producing temporary dental prostheses, but its potential has now expanded to include permanent restorations such as single crowns and inlays/onlays [2].

The materials used in dentistry vary depending on whether the restorations are temporary or permanent, with significant differences in their mechanical and aesthetic properties. Temporary restorations are designed for short-term use, providing adequate durability but are more prone to cracking, fracturing, and wear [3]. In contrast, permanent materials are intended for long-term use, offering superior strength and resistance to wear [4]. As the new resin materials are frequently introduced to the market, it is crucial to conduct investigations on the mechanical properties of these products before their clinical use [5,6].

Flexural strength (FS), an essential mechanical property, defines a material’s ability to resist deformation under load combining compressive and tensile stresses and is a key indicator of fracture resistance of the materials. The International Organization for Standardization (ISO) defines minimum standards for the FS of dental materials: ISO 10477 specifies a minimum FS of 50 MPa for polymer-based temporary restorations, while ISO 6872 requires at least 120 MPa for permanent ceramic restorations [7]. According to ISO 4049:2019, for polymer-based restorative materials (excluding luting materials), the minimum FS depends on the type of restoration: 80 MPa for low-stress restorations and 100 MPa for high-stress restorations.

Additionally, the flexural modulus (FM), which measures a material’s stiffness or rigidity, indicates its resistance to elastic deformation when subjected to bending stresses [8]. While a higher FM enhances the durability of a restoration, it must be carefully balanced to ensure biological compatibility and support of periodontal health. Excessive rigidity can place excessive pressure on gingival tissues, potentially leading to inflammation or recession. Moreover, inadequate adaptability to dynamic chewing forces may also lead to microleakage in marginal areas [9]. Therefore, achieving an optimal balance between rigidity, marginal adaptation, and the biomechanical needs of the surrounding tissues is crucial for successful restorations.

Studies have indicated that the FS and FM of polymethyl methacrylate 3D-printed PMMA-based materials are influenced by various factors, including chemical composition, polymerization method, micro-structure, and usage conditions. Moreover, it has been shown that modifying the printing orientations during the production phase of three-dimensional (3D)-printed resin materials produced by the AM technique directly affects the FS and FM of the materials [6,10,11,12,13,14]. Therefore, recent advancements have focused on improving the mechanical properties of 3D-printed resins by incorporating reinforcing agents such as zirconia, glass fibers, carbon fibers, ceramics, silica, polyether ether ketone (PEEK), graphene, aramid fibers, and titanium [7,15,16,17].

In addition to mechanical properties of the materials, their durability and behavior under intraoral conditions are also critical factors to consider [10]. Artificial aging, which simulates intraoral conditions, refers to the gradual degradation of dental materials due to environmental factors such as moisture, temperature fluctuations, and mechanical stresses. Over time, this degradation can negatively affect mechanical properties of PMMA resins, leading to a reduction in their overall performance [6,7]. To the best of the authors’ knowledge, although the mechanical properties of AM materials have been investigated in the literature, there are a limited number of studies that have investigated their post-aging properties [7,8,9,10,11,12,13,14,15,16,17,18,19]. None of these studies simulated a three-year period.

The aim of this study is to investigate the effects of aging and print orientation on the FS and FM of novel permanent 3D-printed PMMA resins reinforced with nano-zirconia and nano-silica. The first hypothesis is that print orientation affects the FS and FM of permanent 3D-printed PMMA resins. The second hypothesis is that aging weakens the mechanical properties of permanent 3D-printed PMMA resins.

## 2. Results

### 2.1. Three-Point-Bending Test

A resin material for additive manufacturing with different orientation (0°—3D-printed and 0°—3D-printed) and a micro hybrid dental resin composite (3M Z250) were used in this study for flexural strength evaluation. The flexural strength (FS) and flexural modulus (FM) of the samples were calculated by means of a three-point bending test, conducted with a universal testing machine (Shimadzu AGX-5 kN Series, Shimadzu, Japan) at a crosshead speed of 1 mm/min and a distance between the supports of 20 mm in accordance with the ISO 4049:2019 standard. The maximum load was obtained from the testing machine. FS/megapascal and FM/gigapascal were calculated with Formulas (1) and (2):

(1) $$Flexural\;strength(MPa)=\frac{3FL}{{2BH}^{2}}$$

F: maximum load (N); L: distance between the supports (mm); B: width of the specimen (mm); H: height of the specimen (mm).

(2) $$Flexural\;modulus(GPa)=\frac{{FL}^{3}}{{4BH}^{3}d}$$

F: maximum load; L: distance between the supports; B: width of the specimen, H: height of the specimen, d: deflection (mm).

**Figure 1:**
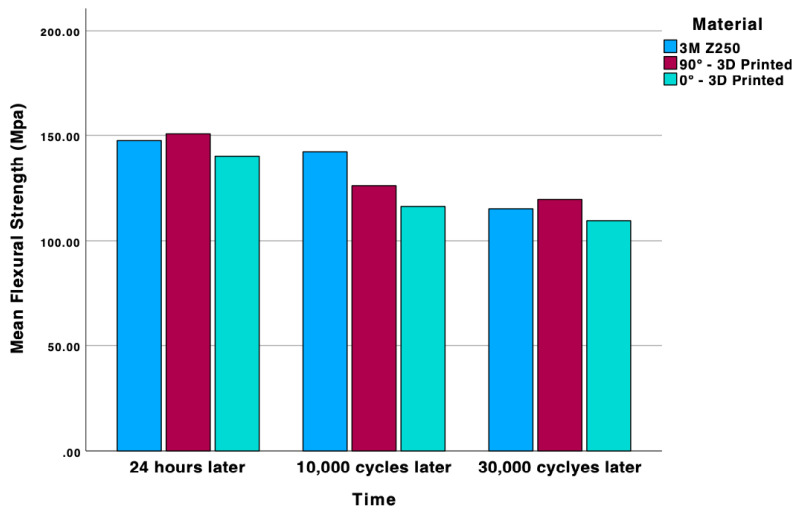
Distribution of Flexural Strength (FS) among different material groups at various aging stages.

**Figure 2:**
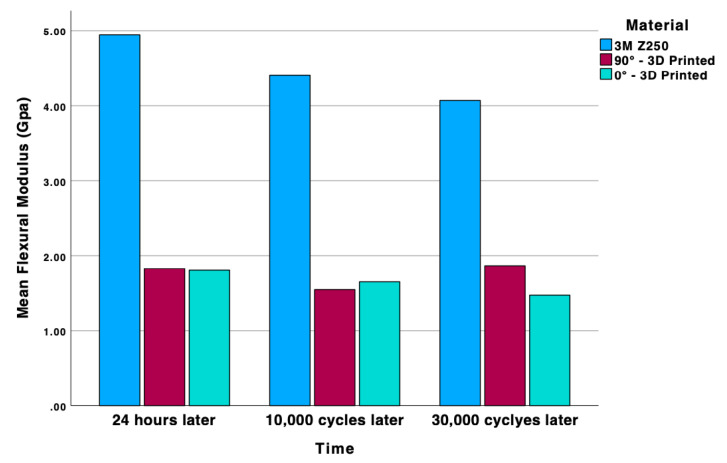
Distribution of Flexural Modulus (FM) among different material groups at various aging stages.

After a 24 h period, the FS values of all materials remained comparable (*p* = 0.125), while a substantial discrepancy existed between the FM and the amounts of deflection (*p* < 0.001 and *p* < 0.001) (Figure 1 and Figure 2). However, after subjecting the materials to 10,000 cycles of aging, significant disparities were identified among the materials in all evaluated parameters (*p* < 0.001). The FS value of micro hybrid composite (142.3 ± 18.88 MPa) was higher than the others. While FS and FM values were comparable, a discrepancy in deflection was observed according to print orientation (2.75 ± 0.371 mm for 90° and 2.37 ± 0.329 mm for 0°). Following the completion of 30,000 cycles of aging, the FS values of the materials exhibited no significant differences. However, the FM value of the micro hybrid composite remained statistically significant compared to the other materials (*p* < 0.001). Furthermore, the degree of deflection exhibited similarity among the various print orientations (Table 1).

An examination of the changes in each material group according to the aging process reveals that the FS value of the micro hybrid composite remained consistent until the conclusion of 10,000 cycles. However, a substantial decline was observed at the end of 30,000 cycles (*p* < 0.001). A decline in FS values was observed for both print orientations after 10,000 cycles for 3D-printed materials (*p* < 0.001). However, after 10,000 and 30,000 cycles of thermal aging, the FS values remained comparable. In contrast to the specimen produced at a 90° orientation angle, a decline in the FS value of the 3D-printed specimen at 0° was evident at the end of 10,000 cycles (*p* < 0.001). The extent of deflection remained constant due to aging in all material groups (Table 2).

### 2.2. Scanning Electron Microscope (SEM) Analysis

After the three-point bending test, the samples were cleaned in an ultrasonic bath with distilled water at 24 °C. The fractured surfaces of one sample from each group were coated with a thin layer of Au/Pd (EM ACE200, Leica Microsystems, Wetzlar, Germany) with 30 s, and examined under a SEM (Zeiss EVO 40, Carl Zeiss, Oberkochen, Germany) at magnifications of 75×, 250×, 1000×, and 5000× with and acceleration voltage of 10 kV.

**Figure 3:**
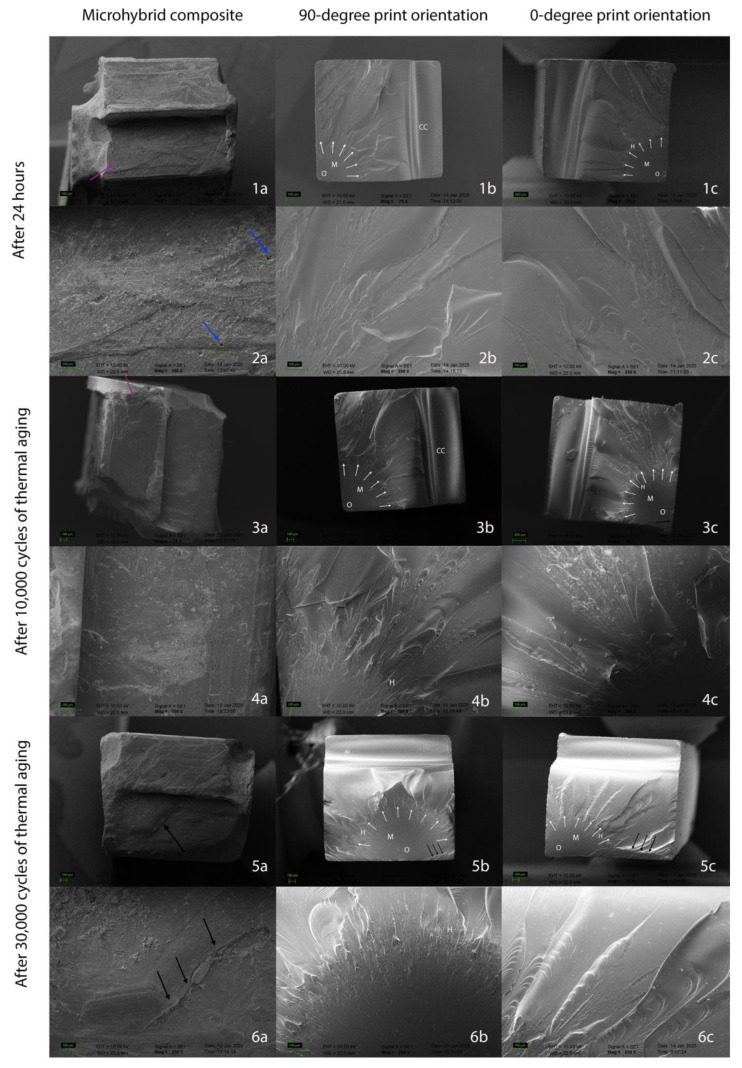
Images 1, 3, and 5 were obtained at 75× magnification, while images 2, 4, and 6 were obtained at 250× magnification with Scanning Electron Microscope (SEM). 1–2. Fracture surfaces of each group after 24 h. 3–4. Fracture surfaces of each group after 10,000 cycles of thermal aging. 5–6. Fracture surfaces of each group after 30,000 cycles of thermal aging. As illustrated in column (**a**), the images depict micro hybrid resin composite. The orientation angle of the 3D-printed resin in column (**b**) is 90°, while the orientation angle of the 3D-printed resin in column (**c**) is 0°. Blue arrows indicate voids from filler detachment. Purple arrows indicate crack formation. White arrows indicate direction of radial crack propagation. Black arrows indicate delamination; (O): fracture origin; (M): fracture mirror; (H): hackle lines; (CC): compression curl.

SEM images revealed irregular fracture surfaces and differences in fracture modes. While the fracture mode remained consistent across different orientation angles in 3D-printed materials, the fracture pattern of the micro hybrid resin composite exhibited distinct characteristics compared to the 3D-printed materials (Figure 3). Within the micro hybrid resin composite group, a predominantly horizontal fracture pattern was observed, whereas 3D-printed resins, regardless of 90° or 0° orientation, exhibited a predominantly radial fracture pattern (Figure 3).

**Figure 4:**
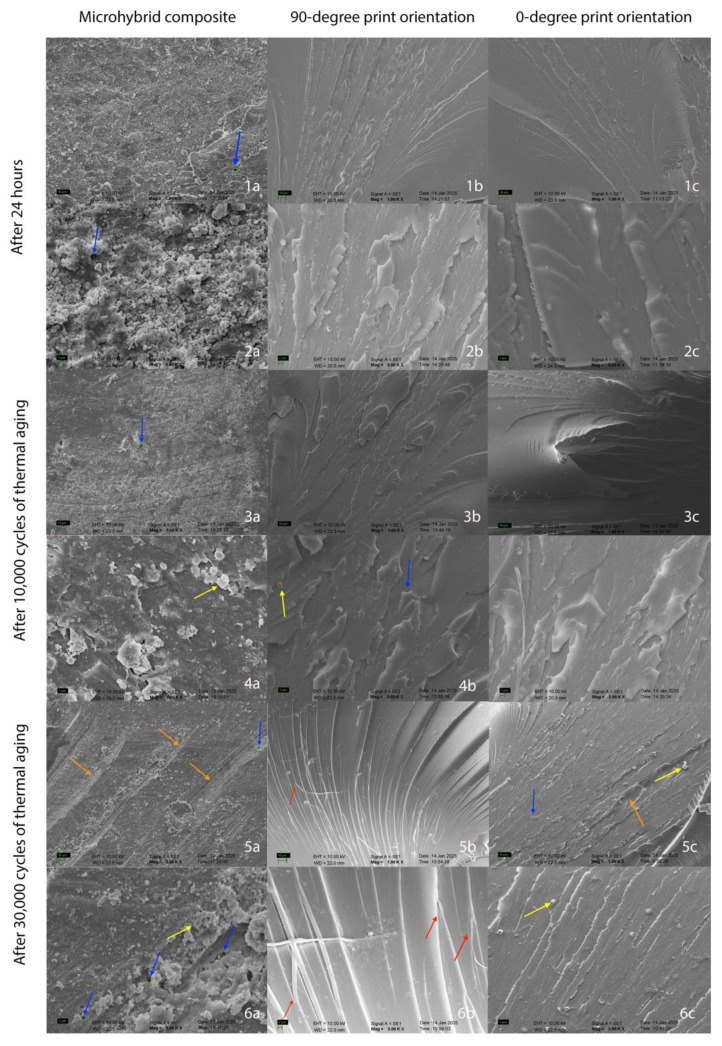
Images 1, 3, and 5 were obtained at 1000× magnification, while images 2, 4, and 6 were obtained at 5000× magnification with Scanning Electron Microscope (SEM). 1–2. Fracture surfaces of each group after 24 h. 3–4. Fracture surfaces of each group after 10,000 cycles of thermal aging. 5–6. Fracture surfaces of each group after 30,000 cycles of thermal aging. Fracture surfaces of each group after 30,000 cycles of thermal aging. As illustrated in column (**a**), the images depict micro hybrid resin composite. The orientation angle of the 3D-printed resin in column (**b**) is 90°, while the orientation angle of the 3D-printed resin in column (**c**) is 0°. Blue arrows indicate voids from filler detachment. Yellow arrows indicate filler particles and clusters. Red arrows indicate the signs of “peeling-like” appearance. Orange arrows indicate the signs of “scraping-like” appearance.

A higher incidence of voids resulting from filler detachment was observed in micro hybrid resin composite compared to 3D-printed resins across all aging durations (Figure 3 and Figure 4, blue arrows). Regardless of orientation, layer thickness was indistinguishable in 3D-printed resins. Furthermore, 3D-printed resins exhibited a more compact and homogeneous microstructure compared to the micro hybrid resin composite at all magnifications. Consequently, filler particles and clusters were more readily observed within the micro hybrid resin composite (Figure 4, yellow arrows). While “scraping”-like surface (Figure 4, orange arrows) appearances were more prevalent on the micro hybrid resin composite, “peeling” appearances (Figure 4, red arrows) were more frequently observed in samples produced with a 90° print orientation.

## 3. Discussion

The findings of this study indicate that print orientation does not have a statistically significant impact on flexural strength (FS) and flexural modulus (FM), while artificial aging significantly affects the FS and FM of 3D-printed zirconia-reinforced PMMA resins. These results highlight that material composition and aging play more important roles than manufacturing parameters such as print orientation. As a result, the first hypothesis of this study was rejected, while the second hypothesis was accepted.

Nanoparticles can be added to 3D-printed resins to enhance their fracture resistance; however, excessive amounts may increase surface roughness, potentially compromising the material’s esthetic and biological performance. In a study by Alshamrani et al., it was observed that the addition of zirconia glass (10% and 20%) and modified glass fillers (5% and 10%) significantly enhanced the mechanical properties of 3D-printed resins compared to the unmodified control group [20]. These findings highlight the role of zirconia nanoparticles in improving fracture resistance.

On the other hand, Salgado et al. [16] demonstrated that increasing the content of graphene-based nano particles beyond 0.1 wt % significantly increased surface roughness in 3D-printed resins. Similarly, in another study by Alshamrani et al. [21], it was shown that surface roughness increased with higher concentrations of zirconia and glass silica fillers. Therefore, when designing resin compositions for long-term applications, it is crucial to consider the balance between mechanical properties, aesthetics, and biological compatibility of the materials. In the present study, a resin material containing 1% organo-modified zirconium oxide and 5.5% organo-modified silicone oxide was investigated with the aim of achieving this balance. However, further studies are required to determine the optimal filler composition and its long-term effects on material performance.

It is well-known that the addition of zirconia to 3D-printed resin materials enhances their mechanical properties [6,20,22]. Additionally, a previous study demonstrated that zirconium oxide improves the sorption and solubility of resin after 3 months [23]. In contrast, the current study found that the flexural strength of the printed resin in both orientations decreased after thermal aging, with a decline observed by the end of the first year. However, no significant difference was noted between the first and third years.

In this study, no statistically significant difference was observed between the FS values of specimens printed at 90° and 0° orientations, indicating that material composition is the primary determinant of FS and FM, while print orientation plays a negligible role. However, some other studies have reported a more significant impact of print orientation on FS. For example, Unkovskiy et al. demonstrated that surgical guide bars (Dental SG, Formlabs) printed at 0°, 45°, and 90° orientations exhibited higher FS values when printed parallel to the build platform (0°) [11]. In contrast, Alghauli et al. conducted a meta-analysis that showed horizontal orientation enhanced FS in certain resin bars, though they also noted variability depending on the design of the device, particularly in bar designs versus occlusal devices [13]. Similarly, Wulff et al. observed that 90° orientation in occlusal device resins resulted in higher FS compared to 0° orientation, highlighting the influence of geometry and material type on the effect of print orientation [12]. Similarly, in the present study, although there was no statistically significant difference, the results indicate that a 90° orientation had a slight positive effect on FS. In another study, no significant impact of print orientation on FS and FM was observed, indicating that print orientation does not have a statistically significant effect on FS [24]. Simeon et al. [24], also reported similar findings, concluding that material composition is the primary determinant of FS, while print orientation plays a minimal role. They also indicated that the influence of print orientation is more pronounced in stereolithography (SLA)-printed materials compared to DLP-printed materials. Considering that the present study utilized a DLP printer, the absence of significant differences in FS across orientations may be attributed to the lower anisotropy typically associated with DLP-printed materials.

In the present study, the FS values of the 3D-printed resin materials were similar after 24 h. The 3D-printed resin composites used in this study contain urethane dimethacrylate (UDMA) monomers, which are associated with high mechanical strength in 3D-printed materials [25]. However, the compared micro hybrid resin composite contains a combination of three monomers—UDMA, BIS-EMA (bisphenol a polyethylene glycol diether dimethacrylate), and BIS-GMA (bisphenol a-glycidyl methacrylate)—which are known for their ability to enhance mechanical properties. Moreover, while conventional resin composite has a micro-filler structure, the 3D-printed resins used in this study possess nano-sized filler. The filler size and content also significantly influence the mechanical properties and durability of the materials [26,27]. However, at the end of 30,000 thermal aging cycles (equivalent to approximately 3 years of intraoral use), the FS of the 3D-printed materials remained lower than that of the micro hybrid composite, while their FS values were comparable. It is important to note that previous studies have emphasized that, in addition to the specified filler and monomer composition, factors such as chemical stability, resin-silane organization, and the polymer network also play a crucial role in influencing the material’s properties [27,28]. In additive manufacturing, post-treatment such as heat or light curing is sometimes required to cross-link unreacted monomers to complete the polymerization process after printing, which improves the final mechanical properties. The amount of polymerization is quantified as the degree of conversion (DC), so mechanical properties and biocompatibility are significantly improved with higher DC [29,30] Therefore, a detailed chemical analysis of the complex organic and inorganic structure of the materials, as well as the polymer network, would be beneficial for future studies. 3D printed materials are subject to viscosity constraints that impede effective filler incorporation, thereby resulting in lower FS [31,32]. It is also necessary to evaluate the following properties: evaluating different filler ratios, alternative post-curing methods, or wear resistance.

Artificial aging caused a substantial reduction in FS across all groups of the current study, with the most significant decline observed after 30,000 thermal cycles (~3 years of simulated aging). This is consistent with previous studies [33,34] showing that hydrolysis, resin matrix plasticization, and filler–matrix interface degradation contribute to reduced mechanical performance. Interestingly, the 3D-printed resins exhibited an earlier stabilization of FS decline after 10,000 cycles (~1 year), unlike the micro hybrid resin composite, which continued to degrade beyond this point. This finding suggests that 3D-printed materials may reach water sorption earlier, thereby mitigating further degradation. A previous study reported that 3D-printed materials showed relatively higher water absorption properties [35].

In a study evaluating the combined effects of different environmental conditions and print orientation, Väyrynen et al. found that vertical specimens exhibited higher FS after wet storage compared to dry storage, particularly in bars printed at vertical, horizontal, and 45° orientations [14]. This study also supports the hypothesis that print orientation-dependent differences in FS may be influenced by environmental factors, such as moisture and storage conditions and aging. Keßler et al. demonstrated that the FS and elastic modulus values decreased with aging [36]. While studies have demonstrated that warm environments can enhance mechanical properties by promoting increased crosslinking [30,33], this study observed a decline in FS. Specifically, a decrease was noted in the micro hybrid resin composite after 3 years and in the 3D-printed resins after 1 year. This finding suggests that factors beyond increased crosslinking, such as material-specific degradation mechanisms or the specific temperature and duration of exposure, may have influenced the observed reduction in mechanical properties. Due to their photosensitive nature, the proper post-curing of these resins is crucial for achieving adequate biological and physical properties [37]. The decline in mechanical properties can be attributed to several factors, including solvent penetration into the resin matrix. Water ingress into the polymer matrix may induce hydrolysis of the organic phase and compromise the interfacial bond between the filler and the matrix, as indicated by previous studies [31,37]. This leads to swelling, plasticization, filler dislodgement or detachment, and the release of unreacted components [33,34], all of which contribute to a reduction in mechanical properties [36]. As a result, the decline in mechanical properties is expected to persist as long as water absorption continues. As suggested by a previous research study [33], stabilization of mechanical properties can be expected once the material reaches saturation. The lack of further decline in FS for 3D-printed resins beyond the first year, unlike the micro hybrid composite, might be attributed to their earlier achievement of water saturation. This observation is further supported by the finding that, although a significant difference in FS existed between micro hybrid composite and 3D-printed resins at the end of the first year of thermal aging, this difference diminished by the third year.

SEM analysis revealed distinct fracture modes between the 3D-printed resins and the micro hybrid composite. While the former exhibited radial fracture patterns, the latter displayed predominantly horizontal fractures, consistent with findings by Keßler et al. [36]. They demonstrated that fracture patterns and surface markings provide characteristic information for materials, and they observed variations in fracture patterns across different materials and aging conditions. In the present study, while SEM analysis revealed no discernible influence of printing orientation or aging on fracture characteristics, a distinct difference in fracture mode was evident between traditional resin composites and 3D-printed resins. Additionally, voids from filler detachment were more prevalent in the micro hybrid composite, as observed in a previous study [10], indicating that aging exacerbates the degradation of resin–filler interfaces. Mudhaffer et al. [10] demonstrated that the 3D-printed samples exhibited evidence of voids resulting from filler detachment after three months. Furthermore, filler particle clustering was prominently observed, and images displayed an absence of filler particles, indicative of peeling. Consistent with these observations, excessive peeling was also observed in the present study. The previous study found no discernible 50 μm layering at 24 h in any of the 0°, 45°, and 90° orientations [10]. Similarly, in the present study when 50 μm layering was used, no distinct layers were observed at any of the orientation stages (0° or 90°). This observation can be attributed to the homogeneity of the structure of 3D-printed resins [32]. The 50 μm layer thickness used in this study is a value recommended in the literature. Borella et al. have shown that a 50 μm layer thickness provides higher flexural strength and hardness compared to 100 μm [38]. Therefore, a 50 μm layer thickness was selected in this study.

The results of this study suggest that aging remains the dominant factor affecting FS and FM, while print orientation has no significant influence on these properties in 3D-printed materials. Clinically, these findings suggest that 3D-printed materials can be suitable for long-term use if material composition and post-processing protocols are optimized properly. Nonetheless, several limitations should be considered. These include the inability to include different print orientations or materials with different content [10], the lack of investigation of other mechanical, optical, and surface properties [39], and the absence of detailed investigation of the polymerization process [40]. Another limitation of the present study is that it evaluates mechanical anisotropy based solely on two print orientations (0° and 90°) using digital light processing (DLP) technology. While this approach allowed for the assessment of directional differences in a clinically relevant and standardized manner, it does not encompass the full spectrum of mechanical behavior across all three spatial axes (x, y, z). Moreover, the influence of print path, slicing strategy, and layer deposition sequence—factors known to affect anisotropic properties—were not systematically investigated. It is therefore recommended that future studies investigate other mechanical and surface properties of these and similar materials, with particular attention to print orientation.

## 4. Materials and Methods

A resin material for additive manufacturing (3D-printing) and a micro hybrid dental resin composite were used in this study with shade A1 (Table 3). For each material, a total of 30 specimens were prepared (N = 30/material), with 10 specimens allocated for each time point: 24 h, 10,000 thermal cycles, and 30,000 thermal cycles (*n* = 10/time point).

### Sample Preparation and Artificial Aging

In the preparation of micro hybrid resin composite samples (Z250, 3M ESPE, Germany) (N = 30/material, *n* = 10/time) with dimensions of 25 (±0.1) × 2 (±0.1) × 2 (±0.1) millimeters, a stainless-steel mold was used according to ISO4049:2019 standards. Each sample was cured for 20 s with an irradiance of 1000 mW/cm^2^ (Valo Cordless, Ultradent, South Jordan, UT, USA) at three distinct locations within a steel mold.

**Figure 5:**
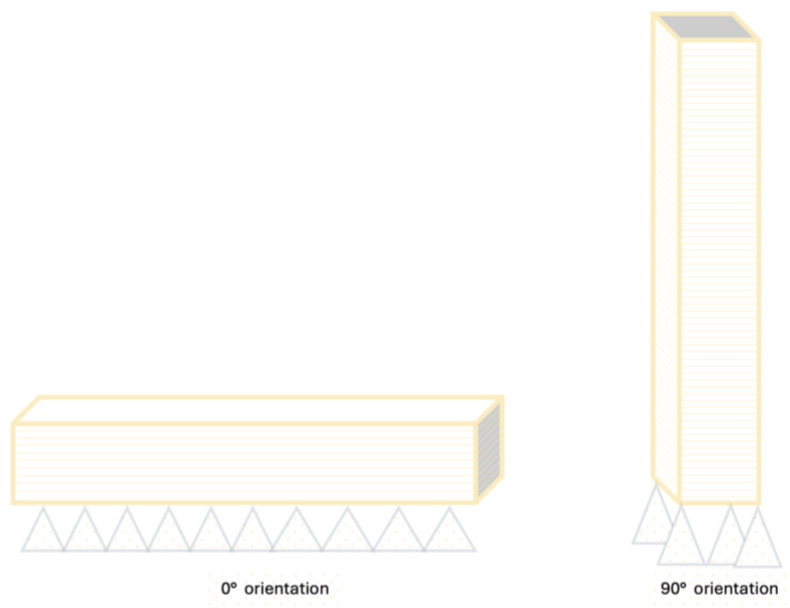
Schematic representation of print design of bar-shaped specimens in 0° and 90° print orientations, respectively.

Samples for the additive manufacturing process (Permanent 3D Printing Polymer Resin, Arma Dental, Istanbul, Turkiye) were printed using a 3D printer (Asiga Max UV, Asiga Inc, Castle Hill, Australia), which employs DLP technology and operates at a light wavelength of 385 nm. The samples were designed using an online software program (Meshmixer, Autodesk, San Rafael, CA, USA) saved as an STL (standard tessellation language) file and imported into the 3D printer’s software program (Asiga Composer, Asiga Inc., Castle Hill, Australia). The samples were then positioned on the build platform in the software at 0° and 90° orientations, enabling the adjustment of different print orientations while maintaining the same dimensions (N = 180/material, 60/orientation). Subsequently, the print parameters were established, including print orientation (0° and 90°) (Figure 5), sample number (*n* = 30/orientation), layer thickness (50 μm), and automatically generated support design. The 0° and 90° print orientations were selected to represent two extreme and commonly investigated angles in additive manufacturing. The 0° orientation reflects printing parallel to the build platform, typically associated with reduced layer interfaces and improved surface quality, while the 90° orientation represents vertical printing, which may increase interlayer boundaries and influence mechanical performance. These two angles allow for a comparative evaluation of the potential anisotropy inherent to the additive manufacturing process.

Following the printing process, the samples were subjected to an automated cleaning procedure utilizing an alcohol-based solution (97% isopropyl alcohol) for five minutes. Then, the samples were removed with a scalpel and post-polymerized for 10 min using 405 nm wavelength curing machines (Lilivis, Huvitz, Anyang-si, South Korea). All surfaces of the samples were polished to a uniform finish using silicon carbide (SiC) papers (400-, 600-, and 800-grit), and the dimensions of the samples were verified with a digital caliper (±0.1 mm).

All samples from each orientation group from Permanent 3D Printing Polymer Resin and Z250 resin composite material were immersed in distilled water at 37 °C for 24 h following the ISO 4049:2019 standard. The remaining materials for each group (*n* = 10 per time point) were subjected to a thermal cycling device (Salibrus Technica, Boston, MA, USA), which was set to a temperature range of 5–55 °C, for a total of 10,000 to 30,000 cycles to simulate the effects of artificial aging. Each temperature was immersed for 30 s, followed by a transfer time of 20 s.

To simulate one year of use in the oral cavity, 10,000 cycles were used to represent thermal aging, while 30,000 cycles were used to approximate three years of use. Consequently, the samples underwent artificial thermal aging and were evaluated after 1 and 3 years of simulated aging [41].

IBM SPSS v29 was used to perform and visualize the statistical analyses. Normality of the data was evaluated using statistical and visual methods, and homogeneity of variances was assessed via Levene’s test (*p* > 0.05). Descriptive data are presented as mean ± standard deviation. For intergroup comparisons of normally distributed data with homogenous variances, a one-way ANOVA was conducted, followed by Tukey’s HSD post hoc test for pairwise comparisons. The Type I error margin was set at 5% (α = 0.05) for all statistical tests.

A post hoc power analysis was performed (by using G*Power) based on the smallest observed effect size (f ≈ 0.38 for flexural strength at 24 h), which revealed a power exceeding 80% at α = 0.05 with *n* = 10 per group.

## 5. Conclusions

Within the limitations of this study, it can be concluded that print orientation does not significantly affect FS in 3D-printed materials, while aging significantly reduces FS, particularly after simulating three years of intraoral use. 3D-printed resins reach stability more quickly under aging conditions compared to micro hybrid composites, while milled resins continue to exhibit superior mechanical properties. These findings highlight the critical importance of material composition rather than print orientation when optimizing the long-term performance of resin dental restorations.

The substantial degradation observed during thermal aging is a critical factor to consider in clinical applications. When planning fixed prostheses, especially in stress-bearing regions, clinicians should prioritize resin materials with proven resistance to aging. Ensuring long-term clinical success will require both the optimization of post-processing protocols and the careful selection of materials with established durability. To build on these findings, further research is recommended, particularly studies that explore additional mechanical properties and variations in material composition.

## Figures and Tables

**Table 1:** Descriptive statistics of flexural strength (FS), flexural modulus (FM), and deflection values by materials groups at different aging stages.

24 h	FS (MPa) Mean ± SD	FM (GPa) Mean ± SD	Deflection (mm) Mean ± SD
3M Z250	147.6 + 16.5	4.94 ± 1.36 ^a^	1.07 ± 0.32 ^a^
90°—3D-printed	150.7 ± 6.2	1.82 ± 0.15 ^b^	2.78 ± 0.30 ^b^
0°—3D-printed	140.2 ± 11.8	1.80 ± 0.21 ^b^	2.63 ± 0.37 ^b^
***p* value** ^1^	0.125	**<0.001**	**<0.001**
**10,000 cycles aged**	**FS (MPa)** **Mean ± SD**	**FM (GPa)** **Mean ± SD**	**Deflection (mm)** **Mean ± SD**
3M Z250	142.3 ± 18.88 ^a^	4.4 ± 1.45 ^a^	1.22 ± 0.526 ^a^
90°—3D-printed	126.1 ± 6.56 ^b^	1.5 ± 0.16 ^b^	2.75 ± 0.371 ^a^
0°—3D-printed	116.4 ± 10.13 ^b^	1.6 ± 0.12 ^b^	2.37 ± 0.329 ^b^
***p* value** ^1^	**<0.001**	**<0.001**	**<0.001**
**30,000 cycles aged**	**FS (MPa)** **Mean ± SD**	**FM (GPa)** **Mean ± SD**	**Deflection (mm)** **Mean ± SD**
3M Z250	115.0 ± 11.8	4.07 ± 0.532 ^a^	0.95 ± 0.13 ^a^
90°—3D-printed	119.7 ± 9.24	1.87 ± 0.842 ^b^	2.38 ± 0.69 ^b^
0°—3D-printed	109.4 ± 6.66	1.47 ± 0.151 ^b^	2.50 ± 0.37 ^b^
***p* value** ^1^	0.068	**<0.001**	**<0.001**

^1^ One-way ANOVA test; there is no statistically significant difference between groups with the same letter (a,b), SD: Standard Deviation.

**Table 2:** Descriptive statistics of flexural strength (FS), flexural modulus (FM), and deflection values of materials by thermal aging.

3M Z250	FS (MPa) Mean ± SD	FM (GPa) Mean ± SD	Deflection (mm) Mean ± SD
24 h	147.6 ± 16.5 ^a^	4.94 ± 1.36	1.07 ± 0.315
10,000 cycles	142.3 ± 18.9 ^a^	4.40 ± 1.45	1.22 ± 0.526
30,000 cycles	115 ± 11.76 ^b^	4.07 ± 0.53	0.95 ± 0.133
***p* value** ^1^	**<0.001**	0.274	0.266
**90°—3D-printed**	**FS (MPa)** **Mean ± SD**	**FM (GPa)** **Mean ± SD**	**Deflection (mm)** **Mean ± SD**
24 h	150.7 ± 6.2 ^a^	1.82 ± 0.15	2.78 ± 0.30
10,000 cycles	126.1 ± 6.6 ^b^	1.55 ± 0.16	2.75 ± 0.37
30,000 cycles	119.7 ± 9.2 ^b^	1.87 ± 0.84	2.38 ± 0.69
***p* value** ^1^	**<0.001**	0.321	0.144
**0°—3D-printed**	**FS (MPa)** **Mean ± SD**	**FM (GPa)** **Mean ± SD**	**Deflection (mm)** **Mean ± SD**
24 h	140.2 ± 8.8 ^a^	1.80 ± 0.21 ^a^	2.63 ± 0.37
10,000 cycles	116.4 ± 10.1 ^b^	1.65 ± 0.12 ^a^	2.37 ± 0.33
30,000 cycles	109.4 ± 6.7 ^b^	1.47 ± 0.15 ^b^	2.50 ± 0.37
***p* value** ^1^	**<0.001**	**<0.001**	0.291

^1^ One-way ANOVA test; there is no statistically significant difference between groups with the same letter (a,b), SD: Standard Deviation.

**Table 3:** Resin materials and their contents used in this study.

Material	Manufacturer	Shade	Type	Composition	
				**Resin Matrix**	**Filler**
Filtek Z250	3M ESPE, Germany	A1	Micro hybrid resin composite	BIS-GMA UDMA BIS-EMA	Zirconia/silica, 0.01–3.5 μm inorganic filler size and 60% filler ratio (volume).
Permanent 3D Printing Polymer Resin	Arma Dental, Türkiye	A1	3D-Printing Resin	TPO, UDMA, acrylic acid ester, bio-based aliphatic urethane acrylate aliphatic urethane methacrylate	1% Organo-modified zirconium oxide (30 nm), 5.5% organo-modified silicone Oxide (50 nm)

Abbreviations, BIS-GMA: Bisphenol A-Glycidyl methacrylate, UDMA: urethane dimethacrylate, BIS-EMA: bisphenol A polyethylene glycol diether dimethacrylate, TPO: Diphenyl (2,4,6-trimethylbenzoyl) phosphine oxide.

## Data Availability

The data contained within the article are available on request from the corresponding author.

## Abbreviations

The following abbreviations are used in this manuscript:

FS	Flexural strength
FM	Flexural modulus
3D	Three-dimensional
PMMA	Polymethyl methacrylate
DLP	Digital light processing
SEM	Scanning electron microscopy
CAD-CAM	Computer-aided design and computer-aided manufacturing
SM	Subtractive manufacturing
AM	Additive manufacturing
ISO	The International Organization for Standardization
MPa	Megapascal
GPa	Gigapascal
PEEK	Polyether ether ketone
SLA	Stereolithography
UDMA	Urethane dimethacrylate
BIS-EMA	Bisphenol a polyethylene glycol diether dimethacrylate
BIS-GMA	Bisphenol a-glycidyl methacrylate
STL	Standard tessellation language
